# Multiple Flap Transfer for Multiple Local Recurrence of Soft Tissue Sarcoma

**DOI:** 10.3390/medicina59081489

**Published:** 2023-08-18

**Authors:** Ryo Karakawa, Hidehiko Yoshimatsu, Yuma Fuse, Tomoyuki Yano

**Affiliations:** Department of Plastic and Reconstructive Surgery, Cancer Institute Hospital of the Japanese Foundation for Cancer Research, 3-8-31 Ariake, Koto-ku, Tokyo 135-8550, Japan

**Keywords:** sarcoma, flap, local recurrence

## Abstract

*Background and Objectives*: Surgical management of local recurrence of soft tissue sarcomas (STS) is still challenging. In this article, we report on multiple flap reconstructions for multiple local recurrences of STS. Their feasibility will be validated by examining clinical cases. *Materials and Methods*: Patients who underwent multiple flap reconstructions for multiple local recurrences of STS between April 1997 and October 2021 were included in this retrospective study. Patient demographics, tumor characteristics, surgical characteristics, and postoperative complications were examined. *Results*: Twenty operations of eight patients were identified. The location of the defects was the back in two, the buttock in two, the groin in two, and the lower extremities in two. The average total number of wide resections was 4.0 and the average total number of flap reconstructions was 2.5. The average follow-up period was 109.4 months. The average size of the defect was 102.4 cm^2^ and the average flap size was 15.7 × 10.8 cm. The histological diagnoses were malignant fibrous histocytoma (MFH) in eight operations, osteosarcoma in two operations, myxoid liposarcoma in two operations, undifferentiated pleomorphic sarcoma (UPS) in six operations, and myxofibrosarcoma (MFS) in one operation. Of twelve subsequent operations, the resection of the previously transferred flap was performed in six operations (50%). The occurrence of take back, flap complications, and donor-site complications in the primary operation group was 25%, 25%, and 12.5%, respectively. The occurrence of take back, flap complications, and donor-site complications in the second and subsequent operation group was 0%, 0%, and 16.7%, respectively. *Conclusions*: Multiple operations including wide resections followed by flap reconstructions for multiple local recurrences are feasible. Reconstructive surgeons should choose the options of the flaps considering the future local recurrence for tumors with a high risk of recurrence.

## 1. Introduction

Soft tissue sarcomas (STS) are relatively rare malignancies that typically occur in the body’s trunk and extremities, representing approximately 1% of all adult malignant tumors [1]. The recommended treatment strategy for STS involves extensive surgical excision ensuring a significant safety margin. This approach often leaves a sizable physical defect. While smaller defects can be addressed with primary closure, larger ones necessitate more advanced plastic and reconstructive surgery techniques, such as local or free flap transfers. These reconstruction techniques have been explored extensively in the academic literature [2,3,4,5,6,7,8,9]. With the current improvements in microsurgical techniques and instruments, some recent studies have shown that free flap transfers have a similar safety profile to the reconstruction using a local flap [2,3,10].

However, even with optimal initial treatments, local recurrences can occur and are associated with reduced overall survival rates [11]. Recurrence rates can range from approximately 6.5% to 25% and are influenced by various factors [12,13,14,15]. Risk factors associated with local recurrence include intrinsic factors, pertaining to patient and tumor features, and extrinsic factors, related to clinical treatment such as adequacy of resection margins and contamination of the operative bed [16]. Many studies have reported that a positive resection margin is associated with increased local recurrence [17,18,19]. However, patients with a negative resection margin also have the possibility of developing a local recurrence. A previous study conducted in the United States demonstrated that 15.2% of patients with STS with a negative microscopic resection margin had local recurrence [18].

Surgical management of local recurrence is still challenging. Although radiation therapy is one option for the treatment of local recurrence, wide resection is still the gold standard. Resection may be necessary for control even in the presence of distant metastasis. Flap transfers are often needed for a large defect after wide resection of the local recurrence. However, there are few reports of multiple operations including wide resections followed by flap reconstructions for multiple local recurrences. In this article, we report on multiple flap reconstructions for multiple local recurrences of STS. Their feasibility will be validated by examining clinical cases.

## 2. Materials and Methods

Institutional Review Board approval was obtained. (#2021-GB-087) This retrospective analysis included a systematic review of all operation reports and clinical follow-up data of patients who underwent multiple flap reconstructions for multiple local recurrences of STS between April 1997 and October 2021. Patient demographic data obtained from the medical records included sex, age, body mass index (BMI), history of smoking, tumor location, number of surgeries, radiation therapy, and follow-up periods. Data on tumor size, histology, defect size, flap size, reconstruction method, and operative time were obtained from operative notes. Soft tissue defects were approximated as ellipses. Data collected during hospitalization included length of stay, operative takeback, flap complications, and donor-site complications. Flap complications included total flap loss, partial necrosis, bleeding, and dehiscence. Donor-site complications included dehiscence, infection, lymphorrhea, and seroma.

### Statistical Analysis

Descriptive statistics were obtained. We compared the postoperative complication rates between the primary operation group and the second and subsequent operation group. We used Fisher’s exact test to compare the proportions of categorical variables, and we used the Mann–Whitney U test to compare the continuous variables. All hypothesis tests had a two-sided significance level of 0.05. All statistical analysis was performed using SPSS v.23.0 (IBM Corp., Armonk, NY, USA).

## 3. Results

### 3.1. Results

In total, 20 operations on eight patients who underwent multiple flap transfers after wide resection of primary or local recurrent sarcoma between April 1997 and October 2021 were identified.

Table 1 shows the patient demographics. There were six males and two females, and their average age at the time of the primary operation was 63.1 (range, 24–77 years). The location of the defects was the back in two, the buttock in two, the groin in two, and the lower extremities in two. The average total number of wide resections was 4.0 and the average total number of flap reconstructions was 2.5. The average follow-up period was 109.4 months.

Table 2 shows a reconstructive summary of all operations. The average size of the defect was 102.4 cm^2^ and the average flap size was 15.7 × 10.8 cm. Of the 20 operations, two (10%) were free flap transfers and eighteen (90%) were pedicled flap transfers. Regarding flap complications, total flap loss which was treated with additional skin grafting was seen in one operation, partial flap loss which was treated conservatively was seen in one operation, flap wound dehiscence was seen in two operations, and infection was seen in two operations. Regarding donor-site complications, surgical wound dehiscence was seen in two operations, and seroma and lymphorrhea were seen in one operation. The histological diagnoses were malignant fibrous histocytoma (MFH) in eight operations, osteosarcoma in two operations, myxoid liposarcoma in two operations, undifferentiated pleomorphic sarcoma (UPS) in six operations, and myxofibrosarcoma (MFS) in one operation. Of twelve subsequent operations, the resection of the previously transferred flap was performed in six operations (50%).

The statistical analysis showed no significant difference between the two groups in the proportion of total complications (50% vs. 33.3%, *p* = 0.65), take back (25% vs. 0%, *p* = 0.15), flap complications (25% vs. 0%, *p* = 0.15), total flap loss (12.5% vs. 0%, *p* = 0.40), partial flap loss (12.5% vs. 0%, *p* = 0.40), flap dehiscence (25% vs. 0%, *p* = 0.15), flap infection (12.5% vs. 8.3%, *p* ≧ 0.99), lymphorrhea (0% vs. 8.3%, *p* ≧ 0.99), donor-site complications (12.5% vs. 16.7%, *p* ≧ 0.99), donor-site dehiscence (12.5% vs. 8.3%, *p* ≧ 0.99), and donor-site seroma (0% vs. 8.3%, *p* ≧ 0.99). There was no significant difference between the two groups in the median operative time (3:40 vs. 3:59, *p* = 0.68) and median postoperative length of stay (27.5 vs. 20.5 days, *p* = 0.79).

### 3.2. Case Reports

#### 3.2.1. Case Seven

**Figure 1:**
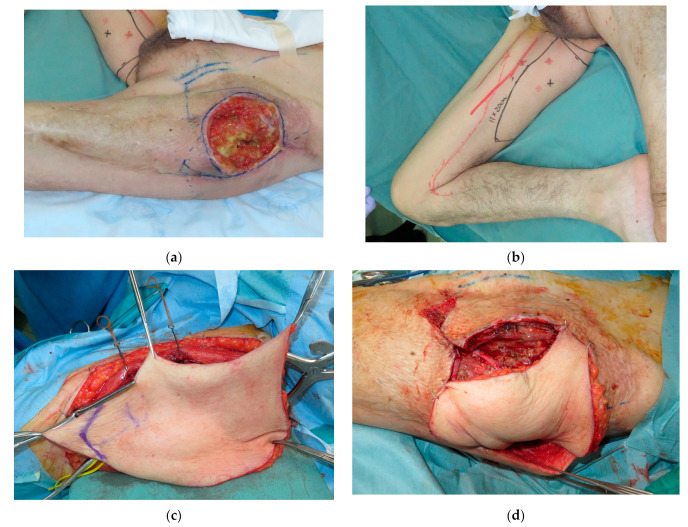

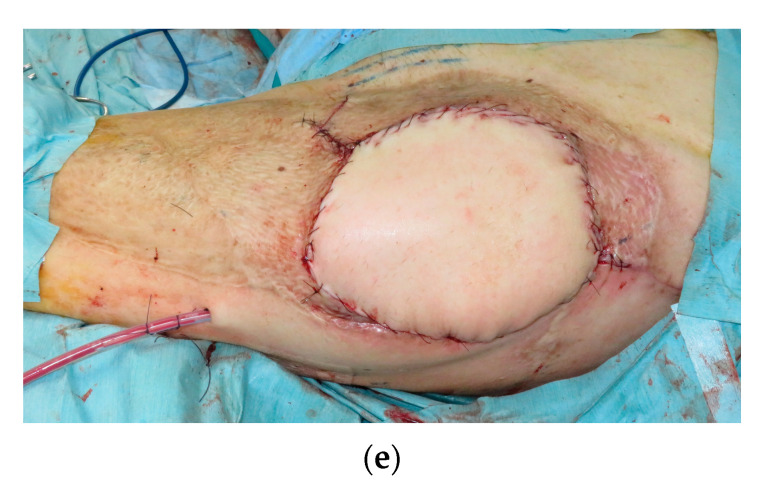
(**a**): The defect after tumor ablation was 11 × 15 cm. (**b**): A 20 × 11 cm profunda femoris artery perforator (PAP) flap was designed. (**c**): The PAP flap was elevated. (**d**): The PAP flap was transferred to the defect. (**e**): Postoperative view.

**Figure 2:**
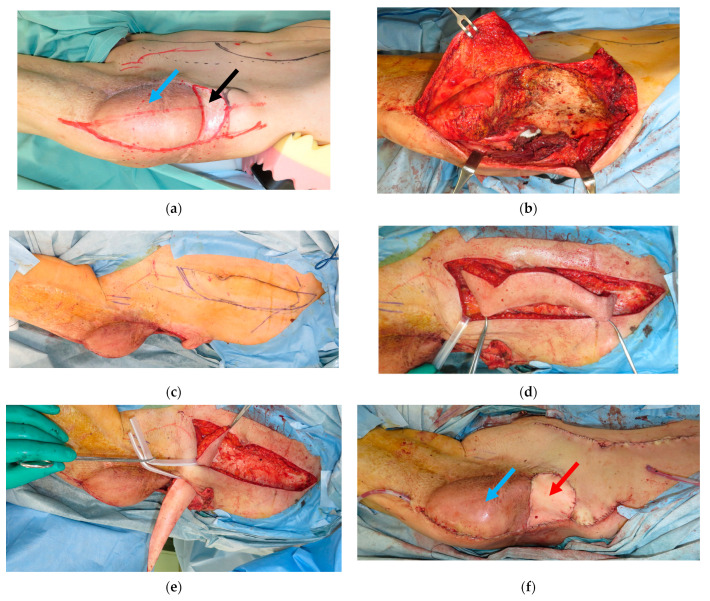
(**a**): A 77-year-old male suffered from a local recurrence of a soft tissue sarcoma on the left groin; black arrow: local recurrence, blue arrow: previously transferred profunda femoris artery perforator (PAP) flap. (**b**): The defect after tumor ablation was 9 × 5 cm and the previously transferred PAP flap was not resected. (**c**): A 5 × 25.5 cm rectus abdominis (RA) flap was designed. (**d**): The RA flap was elevated. (**e**): The RA flap was transferred to the defect. (**f**): Postoperative view; red arrow: RA flap, blue arrow: previously transferred PAP flap.

**Figure 3:**
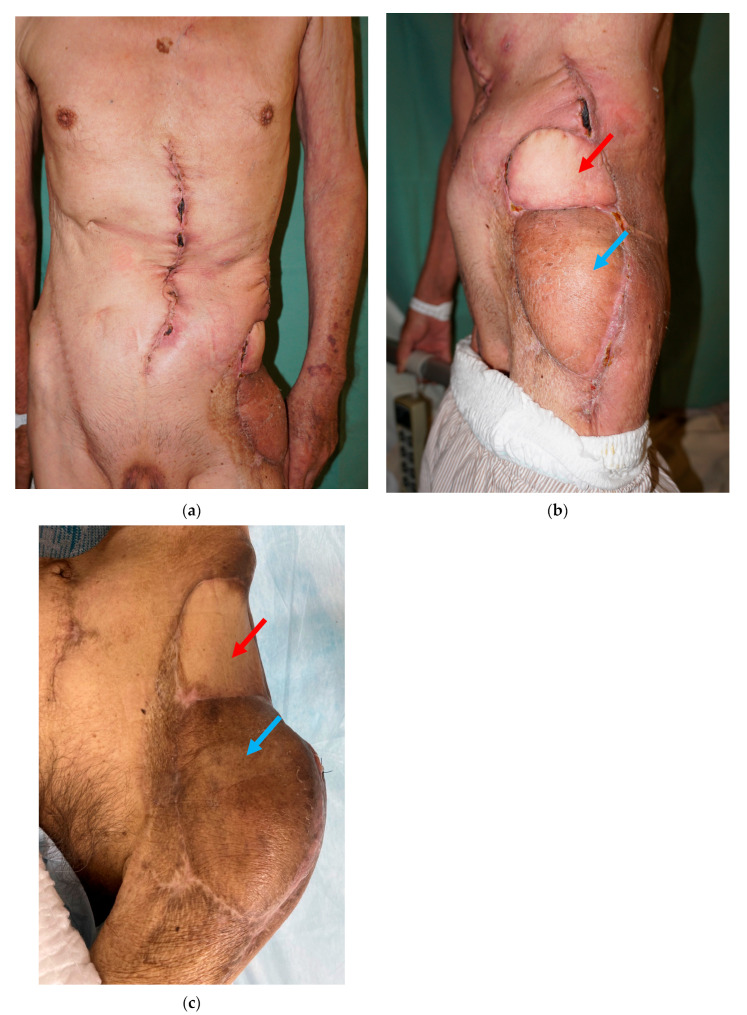
(**a**−**c**) Postoperative view at one month after the surgery; red arrow: rectus abdominis (RA) flap, blue arrow: previously transferred profunda femoris artery perforator (PAP) flap.

A 72-year-old male suffered from a soft tissue sarcoma on the left groin. Surgical wide resection followed by immediate reconstruction using a pedicled anterolateral thigh (ALT) flap from the left thigh was performed. However, the transferred flap was extensively necrotic, and wound healing was achieved with additional skin grafting. Three years later, when he was 75 years old, a local recurrence occurred on his groin. Surgical wide resection with a 1 cm margin including the previous transferred flap was performed. The defect after tumor ablation was 11 × 15 cm (Figure 1a). Then, a 20 × 11 cm PAP flap was elevated from his right medial thigh and transferred to the defect (Figure 1b–e). The pedicle of the PAP flap was anastomosed to the transverse branch of the lateral femoral circumflex artery and vein. All procedures were performed in the prone frog-leg position. Although the flap survived completely, donor-site wound dehiscence was seen, which was treated conservatively. Postoperative radiation therapy with 60 Gy was performed because the margins of the resected specimen showed positive. Moreover, two years later, when he was 77 years old, a local recurrence occurred on his groin again (Figure 2a). Surgical wide resection followed by immediate reconstruction using a pedicled rectus abdominis musculocutaneous (RA) flap was planned. The defect after tumor ablation was 9 × 5 cm (Figure 2b). The previously transferred PAP flap was not resected. A 25.5 × 5 cm RA flap was elevated and transferred to the defect (Figure 2c–f). The flap survived completely, and no postoperative complications were seen during the hospital stay (Figure 3).

#### 3.2.2. Case Eight

**Figure 4:**
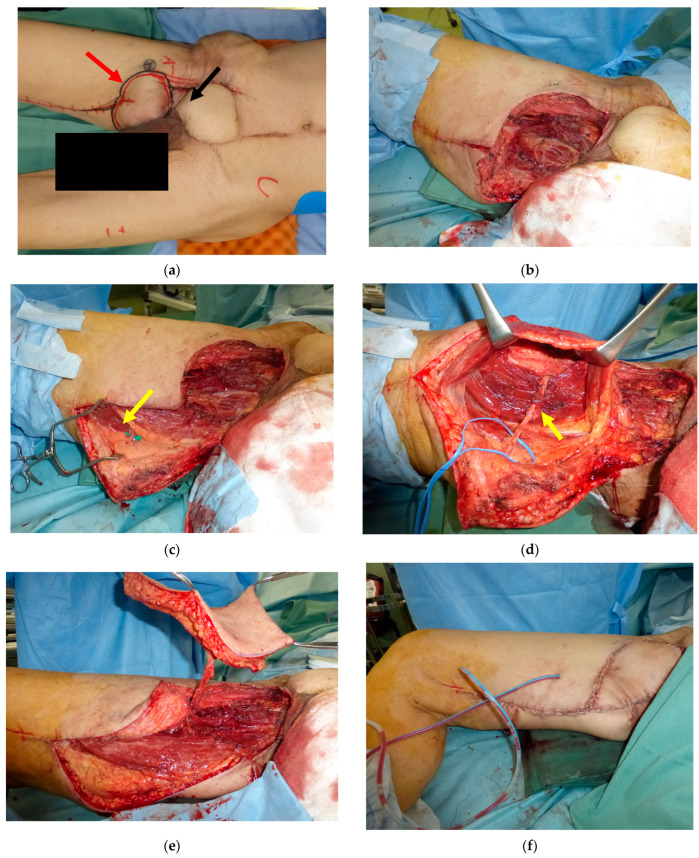

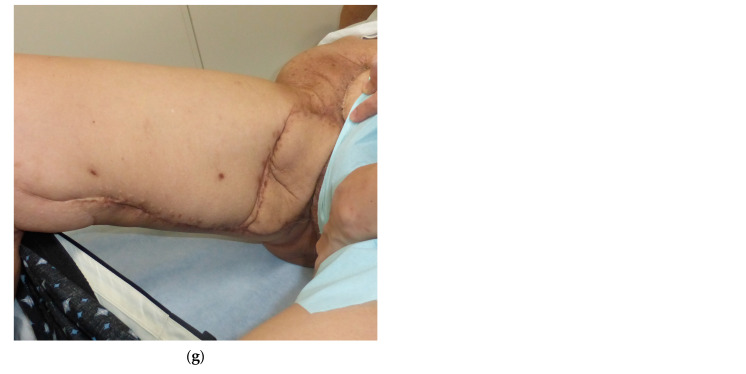
(**a**): A 80-year-old male suffered from a local recurrence of a soft tissue sarcoma on the right groin; red arrow: local recurrence, black arrow: previously transferred rectus abdominis (RA) flap. (**b**): The defect after tumor ablation was 12 × 9 cm. (**c**): A perforator from profunda femoris artery was identified near the defect; yellow arrow: perforator from profunda femoris artery. (**d**): The pedicle was dissected proximally; yellow arrow: perforator from profunda femoris artery. (**e**): A 18 × 9 cm profunda femoris artery perforator (PAP) flap was elevated. (**f**): The PAP flap was transferred to the defect. (**g**): Postoperative view at four months after the surgery.

A 70-year-old male was referred to our department with local recurrence of a soft tissue sarcoma on the right groin. He had undergone the resection of the tumor with primary closure seven years previously and had undergone radiation therapy with 40 Gy for a local recurrence a month previously. Surgical wide resection followed by immediate reconstruction using a pedicled right RA flap was performed. Five years later, when he was 75 years old, a local recurrence occurred on his groin. Surgical wide resection including the transferred RA flap followed by immediate reconstruction using a pedicled gracilis musculocutaneous flap and femoral artery reconstruction using a great saphenous vein were performed. Two months later, lymphorrhea from the right groin and surgical site infection occurred. Then, the debridement followed by pedicled left RA flap transfer was performed. Five years later, when he was 80 years old, a local recurrence occurred again on his groin. (Figure 4a) Surgical wide resection followed by immediate reconstruction using a pedicled PAP flap was planned. The defect after tumor ablation was 12 × 9 cm (Figure 4b). An 18 × 9 cm PAP flap was elevated from the right medial thigh and transferred to the defect (Figure 4c–f). The flap survived completely, and no postoperative complications were seen during the hospital stay (Figure 4g).

## 4. Discussion

We reported our institution’s experience with a flap transfer for reconstruction after resection of STS local recurrence. Our results showed no significant associations in postoperative complications between the first surgery groups and the second and subsequent surgery groups.

The focus of this study was to ascertain the safety and viability of utilizing flap-based reconstructive surgeries for local recurrence during second and subsequent surgical interventions. It’s important to mention that we encountered minor postoperative complications in a small number of cases—four to be exact. However, we managed to address these complications through conservative treatment methods. It’s worth noting here that in order to ensure the safety of subsequent reconstructive surgeries that employ a flap for local recurrence, a meticulous and thorough preoperative assessment is paramount. This assessment should take into account the state of arteries and veins in close proximity to the defect, leveraging tools such as CT angiography or ultrasonography [20,21,22].

Past research in this area provides a roadmap for improving the outcomes of these procedures. For instance, one study proposes combining color Doppler ultrasound and CT angiography to enhance the precision of preoperative perforator navigation [21]. Additionally, the surgeons tasked with these procedures need to have a firm understanding of the available local flap options around the defect, as well as their anatomical properties. This is crucial in order to make informed decisions during surgery. Further, when the use of a free flap is being considered, the surgeon should also be aware of the various flap harvesting methods that can be employed, which are largely dependent on the specific location of the defect [7].

Several studies have addressed the challenge of reconstructions following groin sarcoma resection [23,24]. A noteworthy example is the work of LoGiudice et al., who presented a discussion centered on a 39-case series where patients underwent groin or lower abdominal wall reconstruction. This was achieved using anterolateral thigh (ALT) or rectus abdominis (RA) flaps [23]. Similarly, Miyamoto et al. presented a report on a 12-case series in which complex groin reconstruction was performed using ALT, RA, or latissimus dorsi flaps [24].

In our own case series, we employed a range of flaps for addressing groin defects subsequent to recurrent sarcoma resection. The list of flaps used includes pedicled RA, ALT, profunda artery perforator (PAP), gracilis, and free PAP flaps. Given the wide array of pedicled flap options in the groin region, it is imperative for surgeons to possess a comprehensive understanding of the anatomy and characteristics of each flap. This knowledge is critical in order to treat local recurrences effectively. Despite some postoperative challenges such as the potential for donor-site herniation or bulging, a pedicled RA flap is generally preferred owing to several factors. These include its reliable vascularity, large skin paddle, sufficient tissue bulk, and relative ease of harvest [25,26].

There are numerous published reports that delve into the topic of reconstruction after the resection of gluteal STS or osteosarcoma [27,28]. A review of these reports reveals that the most common flaps utilized are the gluteus maximus (GM) musculocutaneous flap, gluteal artery perforator (GAP) flap, ALT flap, RA flap, gluteal thigh flap, and latissimus dorsi (LD) flap [27,28,29,30,31,32]. For smaller defects, surgeons often turn to perforator flaps such as the GAP. On the other hand, larger defects that require more extensive filling typically employ the GM musculocutaneous flap, RA flap, or free LD flap [27].

In the case series presented in our study, we used pedicled GM musculocutaneous, RA, and ALT flaps for gluteal defects that followed recurrent sarcoma resection. We found no complications resulting from these procedures. The choice of an ALT flap necessitates very detailed pre- and intra-operative planning. This is due to the potential risk of partial necrosis resulting from strong tension on the flap, especially given the significant distance from the buttock to the ALT donor site [28].

A high local recurrence ratio of up to 50–60% of tumors of the type known as MFH has been reported [33,34,35,36,37,38]. Previously, tumors such as myxofibrosarcoma (MFS) and undifferentiated pleomorphic sarcoma (UPS) were classified under MFH. However, they have since been recategorized as distinct entities in the 2002 and 2013 World Health Organization (WHO) classification system for tumors [39]. Furthermore, a past study found a connection between the tail sign on MRI and high local recurrence rates of MFS and UPS [38].

In this study’s 20 operations, we found eight cases (40%) were MFH, one case (5%) was MFS, and six cases (30%) were UPS. Of the second and subsequent operations, resection of the previously transferred flap was necessary in 50% of cases. This was largely due to tumor infiltration, suggesting that the resection should not only address the area surrounding the recurrent tumor but also the previously transferred flap to ensure an adequate resection margin. Consequently, for high-risk recurrence tumors, we believe it’s critical that reconstructive surgeons consider the options of the flaps for future local recurrence even before the initial surgery.

While this study has provided valuable insights, it is important to acknowledge its limitations, most notably, the small number of clinical cases involved. In order to provide a more robust and statistically significant analysis, further research involving a larger number of patients is needed.

## 5. Conclusions

Multiple operations including wide resections followed by flap reconstructions for multiple local recurrences are feasible. Reconstructive surgeons should choose the options of the flaps considering the future local recurrence for tumors with a high risk of recurrence.

## Figures and Tables

**Table 1:** Patient demographics.

No	Age (Year)	Sex	BMI	Tabacco History	Defect Location	Total Number of Wide Resection	Total Number of Flap Reconstruction	Radiation Therapy	Follow-Up Period (Months)
1	77	male	17.8	-	lower extremity	6	2	+	150
2	68	female	21.1	-	buttock	4	2	+	207
3	58	female	23.2	-	lower extremity	4	3	−	168
4	69	male	25.0	-	buttock	4	2	+	18
5	24	male	17.6	-	back	2	2	−	72
6	67	male	20.4	+	back	2	2	−	64
7	72	male	17.3	+	groin	3	3	+	67
8	70	male	26.6	-	groin	7	4	+	129
Median	68.5		20.7			4.0	2		101
IQR	64.8–70.5		17.8–23.6			2.8–4.5	2–3		66–155

Abbreviation: BMI, body mass index; IQR, interquartile range.

**Table 2:** Reconstructive summary.

No	Patient No	Age (yr)	Tumor Size (cm)	Defect Size (cm^2^)	Histology	Margin Positive	Flap Resection	Free/Pedicled	Flap	Flap Size (cm)	Complications
1	1	77	20 × 13 × 12	95.0	MFH	+	N/A	pedicle	GC	15 × 11	
2	1	85	9 × 36 × 29	50.2	MFH	+	+	pedicle	TFL	10 × 8	skin-graft necrosis
3	2	68	35 × 25 × 10	63.4	MFH	+	N/A	pedicle	GM	10 × 9	
4	2	74	25 × 15 × 30	78.5	MFH	−	-	pedicle	ALT	18 × 10	
5	3	58	35 × 25 × 40	84.8	MFH	−	N/A	pedicle	GC	12 × 9	
6	3	61	12 × 8 × 13	28.3	MFH	−	-	pedicle	ALT	13 × 6	
7	3	63	22 × 28 × 30	62.8	MFH	+	+	pedicle	GC	10 × 8	
8	4	69	170 × 78 × 55	129.5	OS	−	N/A	pedicle	RA	17 × 11	
9	4	69	50 × 35 × 18	112.3	OS	−	-	pedicle	GM	15 × 11	
10	5	24	8 × 7 × 4	153.9	myxoid LS	+	N/A	pedicle	LD	29 × 14	donor-site dehiscence
11	5	25	95 × 55 × 28	400.4	myxoid LS	−	+	free	ALT/TFL	22 × 9, 30 × 9	
12	6	67	90 × 85 × 65	213.5	UPS	−	N/A	pedicle	LD	22 × 12	take back, partial flap loss
13	6	68	19 × 14 × 12	50.2	UPS	−	-	pedicle	trapezius	17 × 8	seroma
14	7	72	67 × 45 × 35	122.5	UPS	−	N/A	pedicle	ALT	24 × 12	take back, total flap loss
15	7	75	110 × 50 × 35	129.5	UPS	+	+	free	PAP	20 × 11	donor-site dehiscence
16	7	77	80 × 66 × 46	35.3	UPS	+	-	pedicle	RA	25.5 × 5	
17	8	70	missing	37.7	MFH	−	N/A	pedicle	RA	8 × 8	infection
18	8	75	50 × 22 × 10	31.4	MFS	−	+	pedicle	sartorius	16 × 4.5	infection, lymphorrhea
19	8	76	N/A	84.8	N/A	N/A	+	pedicle	RA	12 × 9	
20	8	80	100 × 75 × 65	84.8	UPS	+	-	pedicle	PAP	18 × 9	
median range		69.5	42.5 × 31.5 × 29.5	84.8						16.5 × 9	
IQR		66–75.3		50.2–124							

Abbreviation: BMI, body mass index; SD, standardized difference; MFH, malignant fibrous histocytoma; OS, osteosarcoma; LS, liposaroma; UPS, undifferentiated pleomorphic sarcoma; MFS, myxofibrosarcoma; GC, gastrocnemius; TFL, tensor fascia lata; GM, gluteal major; ALT, anterolateral thigh; RA, rectus abdominis; LD, latissimus dorsi; PAP, profunda femoris artery perforator; IQR, interquartile range.

## Data Availability

The original contributions presented in the study are included in the article, further inquiries can be directed to the corresponding author.
